# A European interlaboratory trial to evaluate the performance of different PCR methods for *Mycoplasma bovis* diagnosis

**DOI:** 10.1186/s12917-019-1819-7

**Published:** 2019-03-12

**Authors:** Henk J. Wisselink, Bregtje Smid, Jane Plater, Anne Ridley, Anna-Maria Andersson, Anna Aspán, Tarja Pohjanvirta, Nella Vähänikkilä, Helene Larsen, Jonas Høgberg, Adélie Colin, Florence Tardy

**Affiliations:** 1 Wageningen Bioveterinary Research, P.O. Box 65, 8200 AB Lelystad, The Netherlands; 2 Animal and Plant Health Agency (APHA), Surrey, UK; 3 National Veterinary Institute (SVA), Uppsala, Sweden; 4 Finnish Food Safety Authority Evira, Kuopio, Finland; 5 National Veterinary Institute, Technical University of Denmark, Lyngby, Denmark; 6 Anses, Lyon Laboratory, JRU Ruminants Mycoplasmoses, Anses, VetAgro Sup, University of Lyon, Lyon, France

**Keywords:** *Mycoplasma bovis*, Bovine respiratory disease, Real-time PCR, End-point PCR, Culture, Ring trial, Bronchoalveolar lavage fluid

## Abstract

**Background:**

Several species-specific PCR assays, based on a variety of target genes are currently used in the diagnosis of *Mycoplasma bovis* infections in cattle herds with respiratory diseases and/or mastitis. With this diversity of methods, and the development of new methods and formats, regular performance comparisons are required to ascertain diagnostic quality. The present study compares PCR methods that are currently used in six national veterinary institutes across Europe. Three different sample panels were compiled and analysed to assess the analytical specificity, analytical sensitivity and comparability of the different PCR methods. The results were also compared, when appropriate, to those obtained through isolation by culture. The sensitivity and comparability panels were composed of samples from bronchoalveolar fluids of veal calves, artificially contaminated or naturally infected, and hence the comparison of the different methods included the whole workflow from DNA extraction to PCR analysis.

**Results:**

The participating laboratories used i) five different DNA extraction methods, ii) seven different real-time and/or end-point PCRs targeting four different genes and iii) six different real-time PCR platforms. Only one commercial kit was assessed; all other PCR assays were in-house tests adapted from published methods. The analytical specificity of the different PCR methods was comparable except for one laboratory where *Mycoplasma agalactiae* was tested positive. Frequently, weak-positive results with Ct values between 37 and 40 were obtained for non-target *Mycoplasma* strains. The limit of detection (LOD) varied from 10 to 10^3^ CFU/ml to 10^3^ and 10^6^ CFU/ml for the real-time and end-point assays, respectively. Cultures were also shown to detect concentrations down to 10^2^ CFU/ml. Although Ct values showed considerable variation with naturally infected samples, both between laboratories and tests, the final result interpretation of the samples (positive versus negative) was essentially the same between the different laboratories.

**Conclusion:**

With a few exceptions, all methods used routinely in the participating laboratories showed comparable performance, which assures the quality of diagnosis, despite the multiplicity of the methods.

**Electronic supplementary material:**

The online version of this article (10.1186/s12917-019-1819-7) contains supplementary material, which is available to authorized users.

## Background

Bovine mycoplasmoses due to *Mycoplasma (M.) bovis* occur in the majority of countries in the world and infections are associated with a variety of clinical manifestations [1], of which respiratory diseases in calves are the most important in Europe [2]. *M. bovis* is considered to be one of the major emerging pathogens of cattle in industrialized countries threatening livestock production [3] and accounting for significant economic and production losses in the beef and dairy industries [2, 4].

In the absence of effective vaccines [5, 6], the control of *M. bovis* mycoplasmoses relies on good husbandry practices, early diagnosis and efficient antimicrobial treatments [2]. Co-infections with viruses and easy-to-culture bacteria which includes the *Pasteurellaceae* family and which are frequent in bovine respiratory diseases (BRD) [7], may complicate the diagnosis. Furthermore, asymptomatic animals may act as reservoirs, enhancing the disease persistence and spread within herds without clinical manifestations [4, 8]. Hence, diagnostic tools for the detection of *M. bovis* need to be rapid, sensitive and specific.

Awareness of the importance of *M. bovis* in BRD has increased in the past decades although its prevalence might have been underestimated due to the lack of veterinary laboratories routinely monitoring for mycoplasmas. Serological methods, which indicates invasive infection [2] are best used as a herd-level disease diagnostic test, and are not ideal for disease investigations at the animal level as misdiagnosis may occur due to the lag period required for antibody formation or, in contrast, to the high seroprevalence in some cattle populations [9].

For direct diagnosis, conventional microbial culture of *M. bovis* is laborious and time-consuming (up to 7 days) as the bacterium has specific and demanding growth conditions. Furthermore, with limited options for specific selection for *M. bovis* by cultural means, diagnosis typically requires a post-culture identification test as other proven pathogenic or opportunistic mycoplasmas like *M. dispar* or *M. bovirhinis* and *M. arginini*, respectively can be isolated from the same clinical samples [10]. Traditional biochemical or growth inhibition tests used for species confirmation has been replaced by several identification techniques such as sandwich ELISA [11], dot immunobinding membrane-filtration tests (MF-dot) [12] or more recently matrix-assisted laser desorption ionization–time of flight mass spectrometry (MALDI-TOF MS) [13, 14] that demonstrate high specificity, but do not improve the overall sensitivity of the diagnosis since they depend on a cultivation step to isolate the organism.

Hence, PCR-based techniques that allow rapid (i.e. within a few hours), sensitive and specific detection have contributed significantly to an improved diagnosis of *M. bovis*. Today several highly sensitive and species-specific PCRs targeting a variety of genes are used in the diagnosis of *Mycoplasma*-associated diseases for *M. bovis* in cattle herds with BRD and/or mastitis (see review by Parker et al., [15] and Table 1 for an overview). However, few studies are dedicated to the comparison of overall performance of these assays [16]. This study describes the comparison of several PCR methods currently used in six national veterinary institutes across Europe. Three different sample panels were collated and used to assess the analytical specificity, analytical sensitivity and comparability of the different PCR methods, respectively. Results were also compared, when appropriate, to those obtained through culturing. The sensitivity and comparability panels were based on bronchoalveolar fluids (BALFs), artificially contaminated or naturally infected, and hence the comparison of the different methods included the whole workflow from DNA extraction to PCR analysis.

**Table 1:** Overview of PCR methods in literature for detection of *M. bovis*

Method	Characteristics	Targeted gene	Published by
End-point PCR	For detection of *M. bovis* in milk samples and nasal swabs	*oppD/F*	[36]
	For detection of *M. bovis* and *M. agalactiae* in diagnostic samples	*uvrC*	[26]
	Design of a species-specific PCR assay for the identification and differentiation of *M. agalactiae* and *M. bovis*	*polC*	[25]
	Development of a *M. bovis* species-specific PCR assay.	*Vsp*	[37]
PCR-DGGE	PCR followed by denaturing gradient gel electrophoresis (DGGE) fingerprinting. For the simultaneous detection of mixed mycoplasma populations including *M. bovis*.	16S rRNA	[27, 35, 38]
DNA microarray	For parallel detection of 37 *Mycoplasma* species, including *M. bovis*.	23S rRNA; *tuf*	[39]
Real-time PCR	For quantifying the load of *M. bovis* in milk, nasal and conjunctival samples from cattle.	*oppD*	[23]
	For pathogens associated with BRD, including *M. bovis*.	*oppD*	[40]
	For detection of *M. bovis* in diagnostic samples.	*uvrC*	[24, 41, 42]
	For detection of *M. bovis* in bovine milk samples	*fusA*	[43]
	For detection of *M. bovis* in bovine milk and lung samples.	16S rRNA	[44]
	For detection of *M. bovis*, *M. dispar* and *M. bovirhinis* in bronchoalveolar lavage fluid from calves.	16S rRNA	[22]

## Materials

### Mycoplasma isolates, growth conditions and viability controls

The mycoplasma isolates used in the study are listed in Table 2. Isolates were grown in PPLO broth, modified as previously described [17], at 37 °C in 5% CO_2_, except for *M. dispar* and *U. diversum* which were grown in modified Friis medium and Eatons general purpose growth medium, respectively [18]. The length of culture was adapted for each species from 3 to 5 days to reach early stationary phase. To enumerate the *M. bovis* culture used for the analytical sensitivity assay (see hereafter), the number of colony forming units (CFU) per ml was determined by plating spots of 2 μl of serial 10 fold broth dilutions onto PPLO agar plates. After incubation for 5 days, colonies from nonconfluent spots were counted under a stereomicroscope and the mean final cell concentration was determined.

**Table 2:** Identity of 17 Mycoplasma strains used in the ring trial to test for the analytical specificity of *M. bovis* PCR methods

Strain	Number	Strain collection	Source	Country	Origin (sample, year)
*Mycoplasma bovis*	F8065	Anses	[45]	France	Bovine respiratory disease, 2014
*Mycoplasma bovis*	F8127	Anses	[45]	France	Bovine respiratory disease, 2013
*Mycoplasma bovis*	F8428	Anses	[45]	France	Bovine respiratory disease, 2013
*Mycoplasma bovis*	F8619	Anses	[45]	France	Bovine respiratory disease, 2014
*Mycoplasma bovis*	L11480	Anses	[45]	France	Bovine respiratory disease, 2000
*Mycoplasma bovis*	L15711	Anses	[45]	France	Bovine respiratory disease, 2011
*Mycoplasma bovis*	L8905	Anses	[45]	France	Bovine respiratory disease, 1995
*Acholeplasma laidlawii*	PG8 NCTC 10116 or ATCC 23206	Anses	TS	UK	Sewage, 1936
*Mycoplasma agalactiae*	5632	Anses	[46]	Spain	Caprine, joint, 1991
*Mycoplasma alkalescens*	PG51 NCTC 10135 or ATCC 29103	Anses	TS	UK	Bovine nasal cavity
*Mycoplasma arginini*	F9238	Anses	Field strain (unpublished)	France	Respiratory disease, caprine, 2014
*Mycoplasma bovigenitalium*	PG11 NCTC 10122 or ATCC 19852	Anses	TS	Not known	Bovine genital tract
*Mycoplasma bovirhinis*	F11020	Anses	Field strain (unpublished)	France	Bovine respiratory disease, 2016
*Mycoplasma canadense*	275C	Anses	TS	Canada	Prepuce of a bull
*Mycoplasma canis*	PG14– NCTC 10146 or ATCC 19525	Anses	TS	UK	Dog throat
*Mycoplasma dispar*	NCTC 10125	APHA	TS	UK	Pneumonic calf lung
*Ureaplasma diversum*	382B16	APHA	Field strain (unpublished)	UK	Bovine, swab from unspecified site

For viability control of *M. bovis* in the BALF samples, laboratories (Lab) 1 and 4 performed a bacteriological examination for *M. bovis* using different methods. In Lab 1, PPLO agar plates were flooded with 200 μl of each BALF (dilution series for sensitivity and individual BALF for comparability), the excess of culture was removed by pipetting and after 5 days incubation the colonies were observed under a stereomicroscope. If colonies of typical mycoplasma morphology were observed, they were picked and grown in PPLO broth until turbidity was observed. In parallel direct 10 fold dilution series up to 10^− 5^ of BALF in PPLO were prepared and incubated until turbidity. Turbid broths (from picked colonies or dilution series) were analysed for the presence of *M. bovis* or other mycoplasma species using MF-dot tests [12] and/or real time PCR (MPBO50). In Lab 4, BALF samples were cultured directly on Friis medium plates and also one 10 fold dilution was made from the BALF in Friis broth [19]. Plates were incubated for 7 days at 37 °C in 5% CO_2_, and inspected every second day under the microscope for mycoplasma growth. Broth medium was incubated at 37 °C for 3 days. The growth and color change were monitored every other day. All broth cultures were examined for presence of *M. bovis* by *oppD* real-time PCR and suspected samples were subcultured on Friis medium plates as described above. The results of the *oppD* real-time PCR on broth were compared with the results of this PCR assay directly on BALF samples and if there was an increase of Ct values it was concluded that this increase was due to multiplication of viable *M. bovis* organisms. A BALF was considered as culture positive when at least one of the different methods returned a positive result.

### Sample panels

Three different sample panels (I, II and III) were compiled to assess the analytical specificity, analytical sensitivity and comparability of the different *M. bovis* PCR methods, respectively (see hereafter for details of the sample panels).

#### Sample panel I: Analytical specificity on DNAs of mycoplasma strains

The analytical specificity was defined as the ability of the assay to distinguish target from non-target organisms [20]. The inclusion list comprises seven *M. bovis* isolates belonging to different subtypes that used to circulate or are currently circulating in France and other countries [21], while the exclusion list was composed of ten strains belonging to ten non-target mycoplasma species that can be recovered from BRD clinical samples (9/10) or are genetically close to *M. bovis* (1/10, *M. agalactiae)* (Table 2). For each strain, a 20 ml broth culture was grown at 37 °C up to the stationary phase and mycoplasma cells were harvested by a 30 min centrifugation at 9000 *g* and 4 °C. DNA was extracted from pelleted cells either using 3 DNeasy Mini Spin Columns from the DNeasy 96 Blood & Tissue Kit (Qiagen) or the Maxwell® 16 Tissue DNA purification kit (Promega). Each DNA solution was diluted to a 10 ng/μl concentration, aliquoted into anonymized individual tubes (marked from A to Q) for each laboratory and frozen at − 80 °C.

#### Sample panel II: Analytical sensitivity on BALF spiked with *M. bovis*

The analytical sensitivity of the *M. bovis* PCR assays was defined as the ability of the assay to detect the lowest concentration of *M. bovis* in CFU/ml in BALF [20]. A pool of BALFs sampled from specific pathogen free (SPF) calves of 3 weeks old was spiked using an enumerated culture of strain 147,826–1 (VB) (isolated in South of Sweden in 2016 from mastitis milk) in order to get a final 1 × 10^8^ CFU/ml concentration. Ten-fold serial dilutions (*n* = 7) of this mixture were prepared in BALF resulting in a range from 1 × 10^7^ to 1 × 10^1^ *M. bovis* CFU/ml. All dilutions were performed in the same BALF pool, aliquoted afterwards in separate anonymized samples (marked from 1 to 7) for every laboratory and frozen at − 80 °C.

#### Sample panel III: Comparability on BALF samples from the field

For comparison of the results of the PCR methods on field samples, BALF samples (*n* = 21) were selected from a set of BALF from veal calves, sampled between October 2013 and April 2014 and used for the evaluation of a triplex real-time PCR for the detection of *M. bovis*, *M. dispar* and *M. bovirhinis* in BALF [22] and stored since then at − 80 °C. In that study, BALF samples were tested from calves on farms with BRD and were positive for *M. bovis* with Ct values below 30 (in this work BALFs n° 4, 6, 8, 9, 13, 16 and 20), weak-positive with Ct values between 30 and 40 (in this work BALFs n° 1, 3, 14, 15, 18, 19 and 21) or negative with Ct values above 40 (in this work BALFs n° 2, 5, 7, 10, 11, 12, 17). These 21 BALFs were anonymised, aliquoted and sent to each participating laboratory on dry ice. Two laboratories performed bacteriological examination of the BALF samples as described above.

### Participating institutes

Six institutes for Animal Health in six different European countries participated in the *M. bovis* PCR ring trial: the Animal and Plant Health Agency (APHA), UK; the National Veterinary Institute, Technical University of Denmark (DTU), Denmark; the Finnish Food Safety Authority (Evira), Finland; the French Agency for Food, Environmental and Occupational Health & Safety (ANSES), France; the National Veterinary Institute (SVA), Sweden and the Wageningen Bioveterinary Research (WBVR), The Netherlands. The institutes were anonymously listed as laboratories 1 to 6.

### Distribution of samples and ring trial procedures

Samples were randomised and sent anonymized on dry ice to all participating laboratories, which were asked to i) note the date of reception, ii) confirm that samples were still frozen at reception and iii) store the samples at − 80 °C until analysis. A report form was prepared and every laboratory was asked to report results in terms of positive, negative or doubtful for *M. bovis* or, in case of problems with the validation of negative and positive controls, as a run failure. Users of end-point PCR were asked to report the electrophoresis technique used and the quantity of PCR mix loaded. Users of quantitative tests were asked to report the Ct values and also the Ct value of the internal positive control (IPC) of the test. All laboratories were asked to document the platform on which the PCR method was run.

For analysis of samples of panel I, every laboratory was asked to i) defrost the aliquots at room temperature (not longer than 30 min), ii) make a 1/10 dilution of each DNA sample (to be stored at 4 °C before running PCR test), iii) run a PCR on the original DNA sample and on the 1/10 DNA dilutions in duplicate using 5 μl of each DNA sample in the PCR mix. All these analyses had to be performed on the day when samples were defrosted and in the same run.

For analysis of samples of panel II and III every laboratory was asked to i) extract DNA from 200 μl BALF of the entire dilution series of spiked samples (panel II) and from the field samples (panel III) using the method routinely performed in their laboratory, ii) resuspend the DNA in an equal 200 μl volume, iii) use 5 μl of the DNA in their PCR mix and iv) run the PCR test in duplicate.

### Methods used in the laboratory

Five different DNA extraction methods were used by the laboratories participating in the ring trial (Table 3). Labs 1, 3 and 4 used the same technique (spin column, silica-membrane extraction from Qiagen). The other three laboratories (2, 5 and 6) all used a robot with magnetic-particle technology. However the kits and the type of robots varied (Table 3).

**Table 3:** Overview of DNA extraction and *M. bovis* PCR methods used by laboratories participating in the PCR ring trial

	Lab 1		Lab 2			Lab 3	Lab 4			Lab 5	Lab 6
DNA extraction methods											
Method (kit)	QIAamp DNA Mini Kit (Qiagen)		Promega Wizard			QIAamp DNA Mini Kit (Qiagen)	QIAamp DNA Mini Kit (Qiagen)			EZ1 DNA Tissue Kit, (Qiagen)	MagNA Pure LC Total Nucleic Acid Isolation Kit (Roche)
Extraction equipment	QIAamp DNA Mini Kit spin column		Promega Maxwell automated DNA extraction			QIAamp DNA Mini Kit spin column	QIAamp DNA Mini Kit spin column			EZ1 robot, (Qiagen)	MagNA Pure LC Instrument (Roche)
Sample lysis buffer	Buffer AL (Qiagen)		Buffer AL (Qiagen)			Buffer AL (Qiagen)	Buffer AL (Qiagen)			Kit based	“Total NA External lysis” protocol
Sample volume (μl)	200		300			200	200			200	200
Elution volume (μl)	200		300			200	200			100	200
PCR methods											
PCR assay	Real-time	End-point	Real-time	End-point	PCR-DGGE	Real-time	Real-time			Real-time	Real-time
Targeted genome region	*polC*	*polC*	*polC*	*uvrC*	16S rRNA	*oppD*	*polC*	*oppD*	*uvrC*	*oppD*	16S rRNA (V3-V4)
Method (kit)	MPBO50 (Thermo Fisher Scientific)		MPBO50 (Thermo Fisher Scientific)	NA		Jumpstart ready mix (Sigma)	MPBO50 (Thermo Fisher Scientific)	NA		NA	QuantiFast triplex Kit Real Time-PCR kit (Qiagen)
DNA volume (μl)	5	5	1	1	1	1	5	5		2	5
Total PCR volume (μl)	25	25	25	50	50	25	25	22	25	15	20
DNA polymerase	Provided in the kit	GoTaq (Promega)	Provided in the kit	Taq Gold (Applied Biosystems)		Jumpstart ready mix (Sigma)	Provided in the kit	iTaq universal		PerfeCTa qPCR ToughMix (Quanta Bio)	Provided in the kit
Thermocycler	LightCycler 480	Bio-Rad	Stratagene MX3000	Bio-Rad C1000 Touch and iCycler		Rotor-Gene (Qiagen)	Bio-Rad CFX96			ABI 7500 Fast	ABI 7500
Number of cycles	45	45	45	30	45	45	45			45	40
Cut-off (Ct)	37	NA	37	NA		36	37			37	Doubtful 35 < Ct < 40; Positive Ct > 40
Electrophoresis methods											
Method	NA	Qiaxcel	NA	E-Gel™ EX Agarose Gels, 2% (Thermo Fisher Scientific)	NA	NA	NA			NA	NA
Volume per lane (μl)	NA	0.2	NA	20	NA	NA	NA			NA	NA
Reference (if the method has been published)											
	NA	[25]	NA	[26]	[35]	[23]	NA	[23]	[24]	[23]	[22]

Seven different PCR methods were used by the participating laboratories (Table 3). Labs 1, 2 and 4 used the commercial real-time MPBO50 kit from ThermoFisher Scientific which is based on the *polC* gene. Labs 3, 4 and 5 used PCRs based on the detection of the *oppD* gene [23]. However the chemistry and / or real-time PCR kits differed (Table 3). Lab 4 used a real-time PCR based on detection of the *uvrC* gene [24] and Lab 6 used a real-time PCR based on the V3-V4 16S rRNA gene [22]. For amplification, six different real-time PCR platforms were used (LightCycler 480, Stratagene MX3000, Rotor-Gene, Bio-Rad CFX96, ABI 7500 and ABI 7500 Fast).

In addition to real-time PCRs, end-point PCRs (*n* = 2) were also run. Lab 1 used a PCR method based on *polC* [25] whereas Lab 2 used *uvrC* [26] for detection of *M. bovis* and a PCR-DGGE method based on the 16S rRNA gene [27], without primary culture step.

### Statistical analysis

The linear correlation coefficient (*r*), the slope and the amplification efficiency (E) of the PCR methods were calculated between the Ct values obtained in the real-time PCRs and the number of *M. bovis* CFU/ml [28]. Calculations were performed using Microsoft Excel spreadsheets.

## Results

### Analytical specificity using DNAs of mycoplasma strains (sample panel I)

**Fig. 1:**
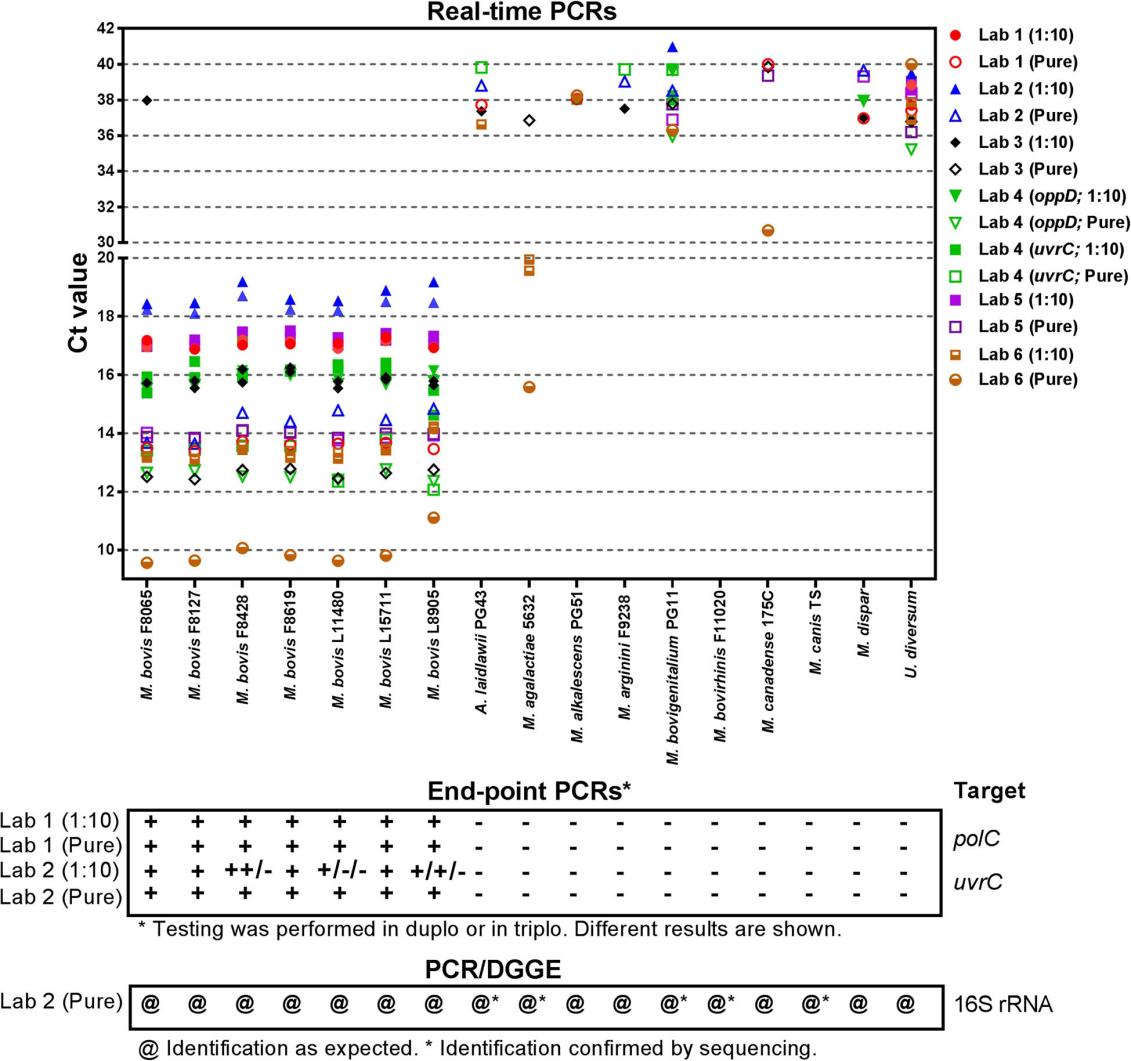
Determination of the analytical specificity of various *M. bovis* PCR assays in use by six laboratories (Lab 1 to Lab 6) on DNA from a panel of seven *M. bovis* strains and 10 non-target mycoplasma species. DNA of each strain was tested in two concentrations: once with 5 μl of 10 ng/μl (pure) and twice with 5 μl of a 1/10 dilution (1:10), except for Lab 4 that used 1 μl of DNA only. Raw data to this figure are provided in Additional file 1

The analytical specificity of the PCR methods was evaluated using DNAs of a panel of target and non-target *Mycoplasma* isolates (sample panel I; *n* = 17). All real-time PCR tests were positive using *M. bovis* DNA extracts except for one test, which was obviously due to an experimental failure (from lab 3) (Fig. 1). Negative PCR results were obtained with the non-*M. bovis* DNAs, except for the 16S PCR by lab 6 which showed a clear positive result on *M. agalactiae* (Fig. 1). Furthermore, several real-time PCR tests of DNA samples from which *M. bovis* was expected to be absent showed weak positive results with Ct values greater than or equal to 37 (Fig. 1). This was especially valid for samples with 50 ng of DNA in the PCR mix, i.e. 5 μl of 10 ng/μl solution. Hence, with most of the PCR assays, Ct ≥ 37 were further regarded as doubtful.

Results from end-point PCRs were in agreement with those of real-time PCRs except for the *uvrC* PCR using 1/10 diluted DNA of *M. bovis* that was poorly reproducible giving alternatively positive or negative results (Fig. 1). The PCR-DGGE analysis fully confirmed the identification of the DNA samples.

### Analytical sensitivity on BALF spiked with *M. bovis* (sample panel II)

**Fig. 2:**
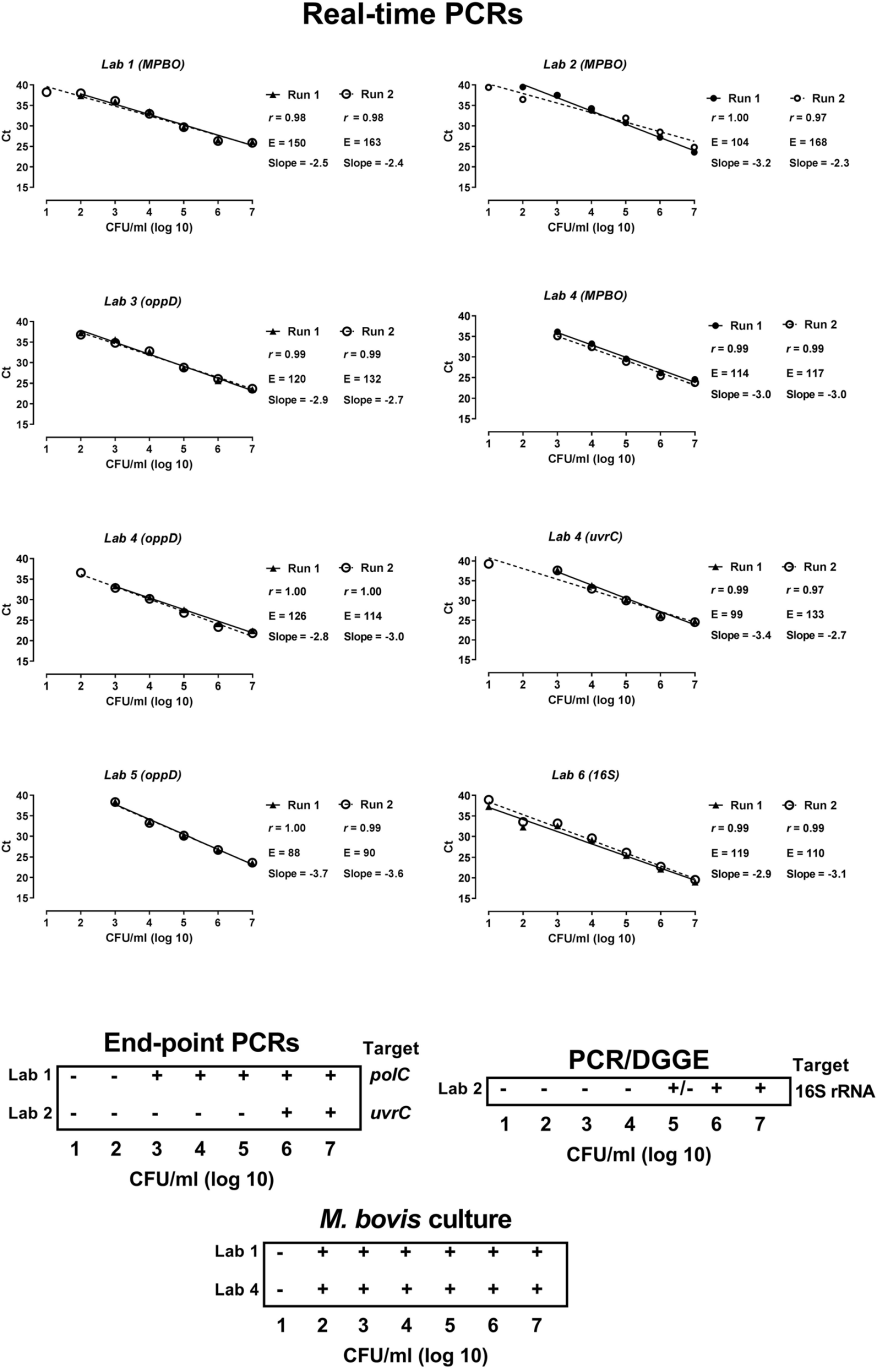
Determination of the analytical sensitivity of various *M. bovis* PCR assays in use by six laboratories (Lab 1 to Lab 6) on spiked bronchoalveolar lavage fluid (BALF) samples. Ten-fold serial dilutions (*n* = 7) of a mixture of 1 × 10^8^ *M. bovis* CFU/ml were prepared in BALF resulting in a range of 1 × 10^7^ to 1 × 10^1^ *M. bovis* CFU/ml and tested by the various PCR methods. Viability of *M. bovis* in the BALF samples was verified with culture. Raw data to this figure are provided in Additional file 1

The analytical sensitivity of the *M. bovis* PCR methods was determined on ten-fold serial dilutions of a pool of BALFs from SPF calves, spiked with *M. bovis* (Sample panel II; Fig. 2). A high linear correlation (*r* > 0.97) between the Ct values and the number of CFU/ml was found for all assays. The slopes ranged from − 2.5 (Lab 1) to − 3.4 (Lab 5) which corresponds to an efficiency (E) of 166 and 97% respectively (Fig. 2).

The limit of detection (LOD) expressed in CFU/ml in the BALF pool varied from 10 to 10^3^ between the different real-time PCR assays. This variation was observed even between laboratories using the same PCR kit (MPBO50, used in Lab 1, 2 and 4) and the same DNA extraction kit (Qiagen, silica-membrane, Lab 1 and 4), suggesting an impact of the PCR platform (LightCycler 480 in Lab 1 versus Bio-rad CFX96 in Lab 4). Similarly, in the PCR method based on the *oppD* gene the LOD was 10^2^ (Labs 3 & 4) and 10^3^ CFU/ml (Lab 5) using different types of DNA extraction methods (Labs 3 &4: spin column; Lab 5: magnetic-particle technology). Detection based on the 16S rRNA gene resulted in a low LOD due to the duplicate copies of the target in the genome (Lab 6). In Lab 4, three different techniques were used on the same extracted DNA batches which allows a classification of the real-time PCR methods as a function of their respective sensitivity, i.e. *uvrC* > *oppD* > *polC*.

The LOD of the end-point PCRs was comparable to that of real-time PCR when targeting the *polC* gene (10^2^ CFU/ml, Lab1) but was higher when targeting the *uvrC* gene (10^6^ CFU/ml, Lab 2) or performing the PCR-DGGE method (10^5^ CFU/ml, Lab 2).

The culture methods used for viability control of *M. bovis* in the BALF samples seemed to be very sensitive since for both laboratories (Lab 1 & Lab 4) the LOD was 10^2^ CFU/ml of BALF.

### Comparability on BALF samples from the field (sample panel III)

**Fig. 3:**
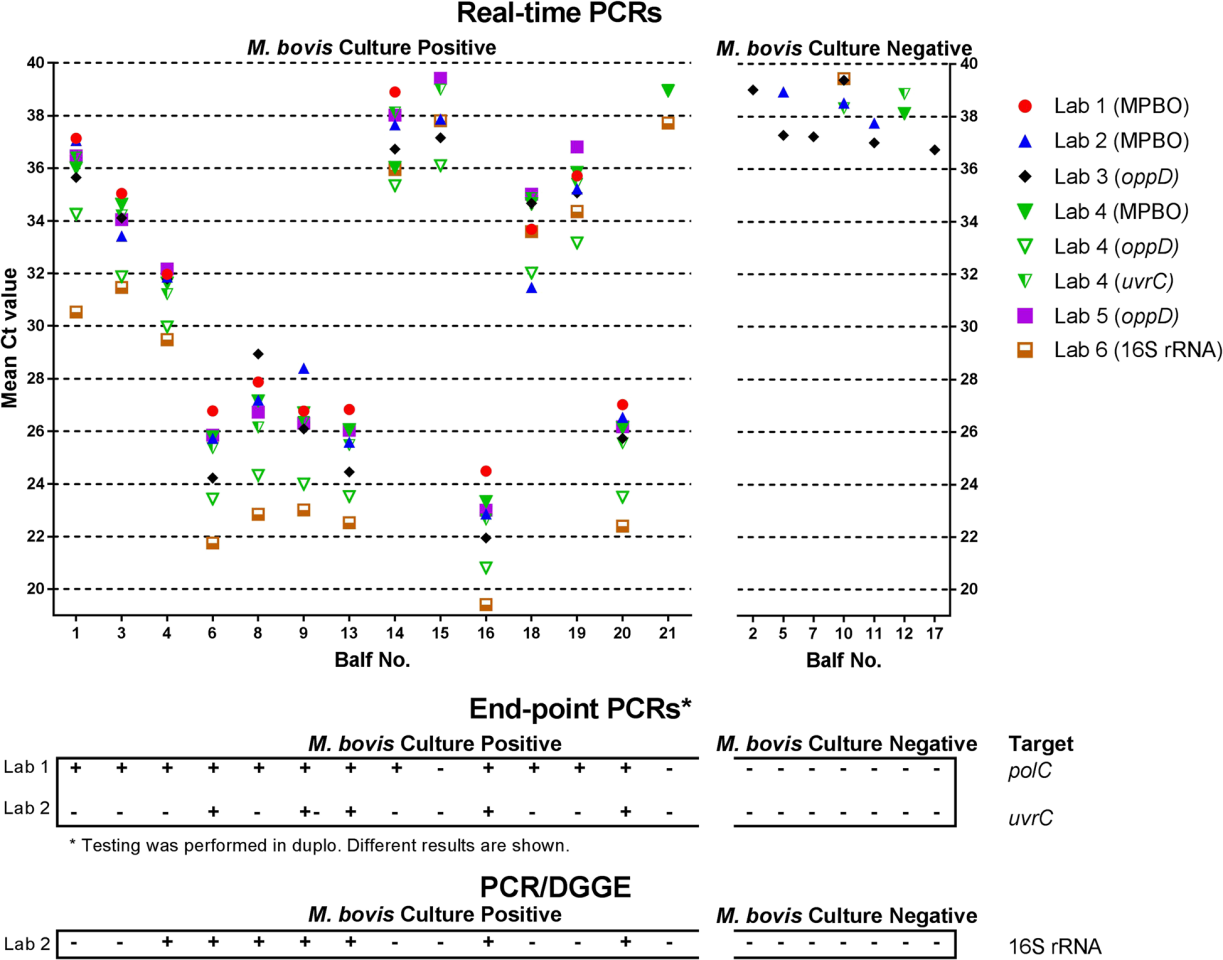
Comparability of results of various PCR methods in use by six laboratories (Lab 1 to Lab 6) on bronchoalveolar lavage fluid (BALF) samples (*n* = 21) from veal calves from farms with bovine respiratory disease (BRD). BALF samples were tested twice by the PCR methods and the mean Ct values were calculated from the real-time PCRs. Viability of *M. bovis* in the BALF samples was verified with culture. On the basis of the culture results, the figure was split in two parts, either with BALF samples negative or positive for *M. bovis* by culture. Raw data to this figure are provided in Additional file 1

A set of 21 BALFs from calves originating from herds with BRD were analysed by PCR in the six laboratories and by culture methods in Lab 1 and 4. Figure 3 shows the mean Ct values (two repeats) obtained by each laboratory. For each BALF, the median dispersion of the mean Ct values was 3.5 but differences reached up to 6.6 Ct values at the maximum. For instance, BALF n°1 had a mean Ct value of 37.1 in the hands of Lab 1 but 30.5 in the hands of Lab 6 (Fig. 3).

Of the seven BALF samples considered as negative (by culture but also by considering previous results from Cornelissen et al. [22], i.e. BALFs n° 2, 5, 7, 10, 11, 12, 17; Fig. 3), none were detected with a Ct < 37. In contrast, of the 14 BALFs that were culture-positive, 11 (79%) were detected by all laboratories with Ct < 37, whereas three (n° 14, 15 and 21; Fig. 3) were weak-positive, doubtful (Ct = 37) or negative at the different laboratories (Fig. 3).

The *polC*-end point PCR gave coherent results compared to the real-time PCR assays in 86% (12/14) culture-positive BALFs but failed to detect BALF n° 15 and 21. In contrast, the *uvrC* end-point PCR and the PCR-DGGE analysis yielded 64% (9/14) and 50% (7/14) false negative results, although the latter test was able to identify also *M. dispar* and *M. bovirhinis* DNA from these samples.

## Discussion

Six laboratories participated in the ring trial and a total of five different DNA extraction methods, seven different real-time and/or end-point PCR methods targeting four different genes and six different real-time PCR platforms were used (Table 2). Among the tested PCR assays there was one ready-to-use commercial kit (MPBO kit, ThermoFisher Scientific); the others were in-house, validated, PCR assays.

Differences in analytical specificity and analytical sensitivity were observed between the various real-time PCR methods. A clear cross reaction was observed in the real-time PCR method of Lab 6 with *M. agalactiae*, which is genetically close to *M. bovis* with a 99% nucleotide similarity between their 16S rRNA sequences [29] but usually not isolated from the bovine host with a few exceptions [30]. Moreover, weak positive results with Ct values between 37 and 40 were obtained on non-target *Mycoplasma* strains which could result in misinterpretation of results and highlights the requirement for thorough assessment of cut-off during validation at individual laboratory level. Differences in the analytical sensitivity appeared to be 10–100 fold between the different real-time PCR methods. Furthermore, in the comparability assay differences in mean Ct values between laboratories appeared to be 6.6 Ct values at the maximum, the median was 3.5 Ct values. A difference of 3.3 Ct values is equal to a 10 fold difference and differences between the real-time PCR methods were therefore 10 to 100 fold, which is comparable to the difference in analytical sensitivity. Nevertheless, this difference did not impact on the qualification of the BALF samples (i.e. positive versus negative), with a few exceptions (Fig. 3). Apparently different real-time PCR methods with a varying sensitivity can be used for the diagnosis of *M. bovis* in clinical BALF samples.

For the present ring trial, BALF from veal calves was selected as a representative clinical sample to compare the efficiency of PCR diagnosis of *M. bovis*. It is estimated that *M. bovis* is a contributing factor in at least 25% of cases of BRD in calves and that BALFs are valuable samples for detection of *M. bovis* in cases of BRD in calves [2, 3, 21, 31]. Other possible samples from BRD cases could include lung tissue samples or swabs from the respiratory tract. However, these would have been less suitable than BALF for homogenous aliquoting and distribution to participating laboratories. Our results should not be generalized to other types of samples, like semen, or milk, or joint fluid/swabs that are increasingly being used for the diagnosis of *M. bovis* [1, 4, 32]. Ring trials based on these types of sample materials warrant further investigation.

The variety of methods in this study is consistent with the number of PCRs described in literature for *M. bovis* detection (Table 1). In order to take into account the specific know-how of each participating laboratory, no technique was imposed upon the laboratories in the design of our ring trial. This is because the focus of the study was to compare the various tests as normally used as evaluated by each laboratory for routine testing. The main consequence of this choice was that the statistical analysis performed in this study was essentially descriptive, with more detailed statistical analysis of the results considered beyond the scope of the present study. While, differences in protocols (such as the number of amplification cycles, the volume of DNA in the mix, etc.) may in part explain the differences in overall performances of the methods, it was not the aim of the paper to analyse these in detail.

In the present ring trial, the detection of *M. bovis* by culture from naturally or artificially contaminated BALFs, as conducted by two laboratories, showed a sensitivity comparable or even better than that of real-time PCR assays. This is in agreement with a previous study that showed no significant difference in the proportion of culture and PCR positive samples [33]. However the authors also stated that the best method to detect and identify *M. bovis* was also dependent on the sample type. Here, the naturally infected BALFs contained several other bacteria [34] including mycoplasmas [22] among which *M. bovirhinis*, which is a known fast grower. Hence, *M. bovirhinis* present in the sample could overgrow *M. bovis* and lead to false negative results depending on the techniques used afterwards to differentiate *M. bovis* from *M. bovirhinis*. The choice of the most acceptable culture medium is also critical and hence diagnosis by culture needs far more expertise than is required to perform PCR, although the latter necessitates good quality DNA and a reliable PCR assay. Of the PCR-based tests investigated in this study only the triplex real-time PCR used by Lab 6 and the PCR-DGGE method used by Lab 2 are able to offer simultaneous identification of three [22] or more *Mycoplasma* species, including hardly cultivable or non-cultivable ones from clinical samples [35]. It should also be noted that PCR-DGGE is most routinely applied in the diagnostic laboratory setting following an initial culture step for clinical sample diagnostics which was not performed here. In the end, the choice of the most suitable method for *M. bovis* diagnosis must reflect the veterinary requirements for a robust and reproducible approach to testing.

## Conclusion

In this study the performance of PCR methods in use by six Animal Health laboratories across six European countries was compared in a ring trial. The analytical specificity of the PCR methods was comparable except for *M. agalactiae*, which was tested positive by Lab 6 because of the use of 16S rRNA as the target gene. The LOD of the real-time PCR methods varied from 10 to 10^3^ CFU/ml and differences in sensitivity to the same order of magnitude were found in the real-time PCR assays on BALF samples of naturally infected veal calves. Despite these differences, and with the exception of the end-point PCR on the *uvrC* gene and the PCR-DGGE method, highly comparable PCR results were obtained on BALF samples from naturally infected veal calves leading to the conclusion that several DNA isolation and PCR methods can give consistent diagnostic test results. Culture results confirmed the presence of viable *M. bovis* in the tested BALF samples, and also confirmed the diagnostic test results of the *M. bovis* PCR methods on these samples.

## Additional file

Additional file 1: Raw data to Figs. 1, 2 and 3 are provided in Additional file 1. (XLSX 29 kb)

## Abbreviations

BALF: Bronchoalveolar lavage fluid
BRD: Bovine respiratory disease
Ct value: Threshold cycle
E: Efficiency
Lab: Laboratory
LOD: Limit of detection
NA: Not applicable
PCR/DGGE: PCR with denaturing gradient gel electrophoresis fingerprinting
*r*: Correlation coefficient
TS: Type strain
